# Multilevel complex interactions between genetic, epigenetic and environmental factors in the aetiology of anomalies of dental development

**DOI:** 10.1016/j.archoralbio.2009.09.005

**Published:** 2009-12

**Authors:** A.H. Brook

**Affiliations:** International Collaborating Centre in Oro-facial Genetics and Development, University of Liverpool, School of Dental Sciences, Edwards Building, Daulby Street, Pembroke Place, Liverpool, L69 3GN, UK

**Keywords:** Dental anomalies, Genetic, Epigenetic, Environmental, Aetiology

## Abstract

Dental anomalies are caused by complex interactions between genetic, epigenetic and environmental factors during the long process of dental development. This process is multifactorial, multilevel, multidimensional and progressive over time. In this paper the evidence from animal models and from human studies is integrated to outline the current position and to construct and evaluate models, as a basis for future work.

Dental development is multilevel entailing molecular and cellular interactions which have macroscopic outcomes. It is multidimensional, requiring developments in the three spatial dimensions and the fourth dimension of time. It is progressive, occurring over a long period, yet with critical stages. The series of interactions involving multiple genetic signalling pathways are also influenced by extracellular factors. Interactions, gradients and spatial field effects of multiple genes, epigenetic and environmental factors all influence the development of individual teeth, groups of teeth and the dentition as a whole. The macroscopic, clinically visible result in humans is a complex unit of four different tooth types formed in morphogenetic fields, in which teeth within each field form directionally and erupt at different times, reflecting the spatio-temporal control of development.

Even when a specific mutation of a single gene or one major environmental insult has been identified in a patient with a dental anomaly, detailed investigation of the phenotype often reveals variation between affected individuals in the same family, between dentitions in the same individual and even between different teeth in the same dentition. The same, or closely similar phenotypes, whether anomalies of tooth number or structure, may arise from different aetiologies: not only mutations in different genes but also environmental factors may result in similar phenotypes. Related to the action of a number of the developmental regulatory genes active in odontogenesis, in different tissues, mutations can result in syndromes of which dental anomalies are part. Disruption of the antagonistic balance between developmental regulatory genes, acting as activators or inhibitors can result in dental anomalies. There are critical stages in the development of the individual tooth germs and, if progression fails, the germ will not develop further or undergoes apoptosis. The reiterative signalling patterns over time during the sequential process of initiation and morphogenesis are reflected in the clinical association of anomalies of number, size and form and the proposed models.

An initial step in future studies is to combine the genetic investigations with accurate recording and measurement of the phenotype. They also need to collate findings at each level and exploit the accurate definition of both human and murine phenotypes now possible.

## 1 Introduction

### 1.1 Aims

The aims of this paper are to review and evaluate current knowledge concerning the aetiology of anomalies of dental development and to propose models to interpret this information as a stimulus for future investigations.

### 1.2 Overview

Dental anomalies are caused by complex multifactorial interactions between genetic, epigenetic and environmental factors during the long process of dental development. This process is multilevel, multidimensional and progressive. It involves multiple interactions and critical stages. In this paper the evidence from animal models, most frequently murine, and from human studies is integrated to outline the current position and to construct and evaluate models, as a basis for future work.

Dental development is multilevel in that it entails molecular and cellular interactions which have macroscopic, clinical phenotypic outcomes. It is multidimensional, requiring developments in the three spacial dimensions, the *x*, *y*, *z* axes and the fourth dimension of time. It is progressive, occurring over a long period, yet with critical stages of development. The series of interactions involve multiple genetic signalling pathways between the ectodermal and the neural crest derived mesenchymal cell layers. The cell signalling is also influenced by extracellular factors. While the term epigenetic can refer to the addition or removal of methyl groups to DNA or the attachment of acetyl groups of histones, here the broader definition of epigenetic is adopted, that is an alteration in gene expression without changes in nucleotide sequencing. This broader view includes interaction between cells at a tissue level as epigenetic events in addition to those directly affecting DNA. At the histological level there are interactions between developing tooth germs.

Interactions, gradients and spacial field effects of multiple genes, epigenetic and environmental factors all influence the development of individual teeth, groups of teeth and the dentition as a whole. The macroscopic, clinically visible result in humans is a complex unit of four different tooth types formed in morphogenetic fields, in which teeth within each field form directionally and erupt at different times. A current, clinically relevant update of the morphogenetic fields concept,^1^ applied by Butler to the mammalian dentition and refined by Dahlberg for the human dentition, incorporates a synthesis with the clone theory^2^ and the odontogenic homeobox code.^3^

Therefore, to evaluate current knowledge of the aetiology of developmental anomalies of the dentition further and to propose aetiological models, the developmental process will be reviewed and then the anomalies will be considered as they occur sequentially in this process: number, size, shape and structure. The conclusions from the evaluation will be based on the structure of the above initial overview and emerging issues for future studies will be outlined.

## 2 The development of the dentition – molecular and cellular interactions

### 2.1 Molecular and cellular interactions – overview

**Fig. 1:**
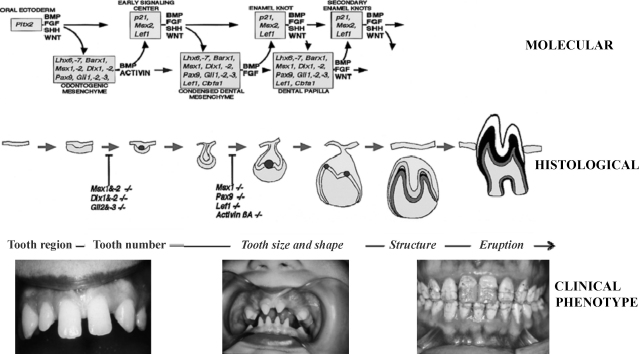
The multilayered developmental process.

During this multilayered developmental process the temporal relationships between the layers as indicated in Fig. 1 are important, as are the critical periods determining whether the process will continue or cease at a particular point for individual teeth or groups of teeth.

Over 300 genes have been identified as involved in dental development,^4^ many of which have functions in cellular communication. Identified multigene signalling pathways, including Fgf, Bmp, Shh, Wnt and Tnf, mediate sequential and reciprocal interactions between the ectoderm and mesenchyme and regulate key transcription factors. In addition to these intracellular links, extracellular effects are illustrated by the low-density receptor-related protein Lrp 4 modulates extracellular integration of cell signalling pathways in development.^5^ This series of reciprocal interactions between factors in the ectoderm and mesenchyme regulates initiation (tooth region and number), morphogenesis (tooth type, size, shape including dimensions and cusp number) and differentiation (tooth structure – enamel and dentine formation and mineralisation).

Mutations in developmental regulatory genes are related to dental development defects.^6^ At a number of stages of the long developmental process repeat signalling sequences are observed^7^ (Fig. 1). Clinically identified associations between some anomalies in humans may well be related to the reiterate nature of parts of the process, as considered further below in the section on anomalies of number, size and form.

In other tissues in the foetus similar interactions, involving many of the same genes, occur in developmental processes. Signalling molecules and growth factors such as Tgf8, Fgf, Shh and Wnt, as well as transcription factors mediating the signals to the nucleus and regulating gene expression, are involved. BMP7 is a central mediator of epithelial–mesenchymal interactions that are necessary for the correct development of structures belonging to the oro-facial complex.^8^ These common developmental factors between dental and other tissues may be reflected in the presence of dental anomalies in clinical syndromes, e.g. ectodermal dysplasia, when mutations occur in genes acting in multiple developmental processes.

Epigenetic factors also play a critical role in tooth development with, for example, histone demethylase regulating dental stem cell differentiation.^9^ Spatial epigenetic information present at each stage of the developmental process, influences later development. Cells can generate patters during development by autonomous mechanisms, by inductive mechanisms involving interaction between cells and by morphogenetic mechanisms.

### 2.2 Initiation

Thickening of the oral epithelium takes place at specific sites to form dental placodes. In determining tooth region within the dental lamina, Fgf and Bmp influence the location of mesenchymal expression of Pax9, a paired box transcription factor.^10^ Pax9 is stimulated by Fgf8 and inhibited by Bmp2 and Bmp4. This antagonistic signalling may determine the site of tooth buds^11^ but Pitx2 and Shh are also present at this stage and tooth germs still develop in the same locations in Pax9 knockout mice.^7^ The Dlx homeobox genes are also important in early patterning of the dental field.^7,12–14^ In addition, the Bmp antagonist Wise and the Wnt co-receptor Lrp 4 provide 5 extracellular communication between mesenchymal and epithelial cells based on the integration of Wnt and Bmp pathways during regulation of tooth number.^5^ At the initiation stage Wise expression is observed in tooth mesenchyme and Lrp 4 in tooth epithelium. At the bud stage Lrp 4 becomes restricted to the epithelial cells at the tips of the bud, while Wise is absent from this area but expressed elsewhere in the tooth epithelium and mesenchyme.^5^

Transcription factors in the Msx, Dlx and Lhx families are necessary to initiation and progression beyond the initiation stage.^4^ Epithelial signals regulate expression of the transcription factors Msx1, Pax9 and Runx2. Msx1 is induced by Bmp and Fgf; Pax9 and Runx2 by Fgf.^4^ Bmp 4 and Msx 1 regulate one another in a positive feedback loop in dental mesenchyme.^15,16^ If any one of these transcription factors is absent in knockout mice, tooth development may arrest at the bud stage^17,18^ but different members of the same family, e.g. Msx1 and Msx2, may compensate for each other when one is inactivated. Msx 1 and Osr 2 act antagonistically in the patterning of the tooth morphogenetic field by controlling the expression and spatial distribution of mesenchymal odontogenic signals along the bucco-lingual axis.^18^ The mouse mandibular first molar tooth germ can inhibit second molar development and an inhibitory cascade model in which initiation of posterior molars depends upon a balance between intermolar inhibition and mesenchymal activation to determine sequential molar initiation has been proposed.^19^

### 2.3 Morphogenesis

In early tooth morphogenesis the critical role of timing is evidenced in the interaction of Pax9, Msx1 and Bmp4. During invitation Pax9 is not a transcriptional regulator of Msx1 and at E12.5 in the mouse Pax9 cannot induce Msx1 expression in dental mesenchyme.^20^ However at E13.5 Pax9 begins to induce Msx1 expression and then Pax9 and Msx1 proteins can induce Bmp4 expession.^20^

During these early tooth morphogenetic events, the Dlx genes are involved in the epithelial–mesenchymal reciprocal signalling events.^21^ The transition from the bud to the cap stage is related to the induction of the enamel knot which expresses signal molecules from the TgfB, Fgf, hedgehog and Wnt families. Shh is an epithelial signal necessary for proliferation which appears to stimulate formation of a reciprocal signal from the mesenchyme back on the epithelium.^22^ Mesenchymal Bmp4 regulates the arrest of the cell cycle in the enamel knot, while Wnts are required for Fgf4 expression.^4^ Fgfs and their receptors are expressed in both epithelium and mesenchyme, regulating proliferation in the adjacent tissue.^17^ The runt domain transcription factor Cbfa1 mesenchymal gene is also expressed during the transition from bud to cap stage.^23^

As tooth morphogenesis advances, the primary and secondary enamel knots, control the development of crown dimensions and cusp formation. The repeated activation and inhibition of signalling is related to differential growth and folding within the tooth germ and determines dimensions and cusp pattern. Enamel knot cells express Fgfs in the dental epithelium regulating growth and folding. The shape of the tooth crown results from this morphogenesis during cap and bell stages when there is rapid proliferation of cells related to folding of the epithelium to form cusp shapes.^7^ While the enamel knot expresses growth stimulatory signals, its cells remain non-proliferative.^24^ These non-dividing cells stimulate proliferation of both the surrounding cells and the mesenchymal dental papillae.^25^ Lpr 3 has restricted expression in the primary enamel knot while Wise is expressed in the mesenchyme at this stage.^5^

In the enamel knots, apoptosis has been suggested as a mechanism controlling the duration of signalling.^24,26^ The expression of Bmp4 in the enamel knot cells is associated with their apoptosis. ^7^ Apoptosis in the enamel knot begins to influence its function as a signalling centre in the late cap to early bell stages.^27,28^ Cessation of activity in the enamel knot is linked to the expression of the cyclin-dependent kinase inhibitor p21 induced by Bmp4. At the early cap stage inhibition of apoptosis does not disrupt the cell proliferation level when compared to controls.^29^ However, the macroscopic morphology shows major differences after inhibition of apoptosis. Mesio-distal diameter is increased and crown height reduced related to the concentration of Z-VAD-fmk treatment; no changes in bucco-lingual dimensions occur. Thus apoptosis in the enamel knot plays an important role in regulating tooth size and shape.^29^

Having undergone apoptosis at the late cap stage, the primary enamel knot is no longer detected at the bell stage. Secondary enamel knots develop at the sites of cusps in teeth with multiple cusps. They produce signalling molecules stimulating proliferation of nearby cells leading to folding of the inner enamel epithelium and subsequent cusp formation.

By this stage the presence or absence, the size and shape of the individual tooth has been determined. The signalling factors which determine tooth region and tooth type are much the same.^30^ The reiterative signalling at the different stages of initiation and morphogenesis may explain the association of differences in tooth size and shape seen in individuals with abnormal tooth number. The occurrence of anomalies, e.g. hypodontia, at different sites around the dentition also relates to alterations in these same signalling processes in all teeth.

### 2.4 Differentiation

At the bell stage when the underlying cusp pattern has been established, the enamel and dentine forming cells differentiate. Initially the odontoblasts secrete the dentine matrix and subsequently the adjacent epithelial cells differentiate into ameloblasts and secrete enamel matrix. The odontoblasts and ameloblasts then control the phases of mineralisation of enamel and dentine. Pleiotrophin (PTN) is expressed in both odontoblasts and ameloblasts and has a critical role in dentinogenesis.^31^

The dentine matrix is predominantly collagen, but also contains other proteins which are mainly products of the Dspp gene present in odontoblasts. Tgf-B signalling controls Dspp expression during odontoblast maturation, with overexpression of TgfB1 causing decreased expression of Dspp.^32^ The Dspp gene is cleaved to produce Dpp, Dsp and Dgp.^33^ Dpp is cleaved off by Bmp1, while Dgp is cleaved by Mmp2 and Mmp20, multiple Mmps being found in dentine. Dpp may act as a nucleator of hydroxapatite formation.^34^ Dlx3 influences morphogenesis and patterning^35^ and in dentinogenesis Dlx3 is expressed during dentine morphogenesis and histodifferentiation and is also present in ameloblasts.^36^ In pre-odontoblast cells Bmp2 regulation of Dspp transcription is mediated by the Dlx3/Osx signalling pathway.^37^

The histone demethylase Jmjd3 regulates the odontogenic differentiation of dental stem cells through an epigenetic mechanism. Jmjd3 knock-down in dental stem cells by small interference RNA results in decreased expression of extracellular dentine matrices. Jmjd3 is essential for the gene expression of homeobox and bone morphogenetic proteins by modifying methylation.^9^

During the initial stages of amelogenesis Dspp also influences enamel hardness, contributing to the structural properties of the layer of enamel adjacent to the amelo-dentinal junction.^38^ In the secretory stage the enamel protein matrix deposited by the ameloblasts is predominantly formed of amelogenin (85%). At the mid-secretory stage for appositional crystal growth and structural maintenance amelogenin is essential.^39,40^ However, while enamelin contributes less than 5% of the matrix it plays a major role in controlling the initiation of hydroxyapatite formation in early amelogenesis,^41^ being necessary for creating and maintaining enamel crystallite elongation at the mineralisation front immediately adjacent to ameloblasts.^42^ The further enamel protein ameloblastin is a cell-adhesion molecule that maintains the differentiation stage of secreting ameloblasts and controls their secretion.^25^

The subsequent breakdown and removal of matrix proteins by means of proteolytic processing is essential for further development and mineralisation. Enamelysin (Mmp20), a matrix metalloproteinase, and Kallikrein (Klk4), a serine protease, are two major molecules involved in this process. Mmp20 is expressed in secretory stage ameloblasts and also has effects on the maturation stage as well as on the mineralisation of mantle dentine.^43,44^ Klk4, present in both ameloblasts and odontoblasts, is expressed at the enamel transition and maturation phase.^45^ Amelogenin is cleaved by Mmp20 and later degraded during maturation by Klk4.

Within the ameloblasts Dlx3 and Dlx6 are expressed throughout the presecretory, secretory and maturation stages. During secretion Dlx2 is switched off and Dlx1 expression is upregulated.^46^ The Dlx homeobox genes may influence enamel formation by the regulation of amelogenin expression.^46^

Normal enamel thickness may be achieved by Runx2 suppressing enamel protein expression at the end of the secretory stage to give normal enamel thickness.^47^ In the maturation phase Runx2 induces Klk4 and upregulates basal membrane protein expression to induce ameloblast attachment to the enamel matrix.^47^

Histodifferentiation for root development follows. The odontogenic epithelium extends apically and odontoblasts and cementoblasts develop on its inner and outer surfaces, respectively. Formation of the root dentine and the cementum follows. Fibroblasts and osteoblasts arising from the dental follicle and the supporting and eruption mechanism of the periodontium are formed.

## 3 Clinical outcomes

### 3.1 Dental anomalies – overview

The outcome of the developmental process is the dentition seen as the phenotype. Valuable findings have emerged from animal as well as human studies in relating phenotype to genotype. Therefore where relevant the findings from animal models will be considered in this section alongside the human studies, while acknowledging the limitations of extrapolating from the animal models.

The human clinical phenotype reflects the progressive nature of the developmental process with different teeth of a given tooth type forming and maturing at different times, with the time gradient following the spatial gradient from mesial to distal of each tooth region. Therefore within a given tooth type there will be teeth at different developmental stages. Similarly, between different tooth types there will be some overlap in development, but also different developmental stages at a given point of time. The complexity of these time and space parameters of dental development is reflected in the complexity of the clinical phenotypes of dental anomalies of number, size, form and structure.

These phenotypes will be considered in the order of the stages of the developmental process at which they have occurred. In keeping with the multilayered nature of the process, the clinical outcome needs to be related to evidence of the tissue changes and to the molecular genetic–epigenetic–environmental interactions.

In addition, the multidimensional outcomes for each tooth reflect more than the influences of the molecular factors that would determine it if each tooth developed in isolation. The interactions of the developing tooth germs for nutrition and space,^1,48^ reflected in their position in morphogenetic fields affect the clinical phenotype.

### 3.2 Anomalies of number, size and shape occurring during initiation and morphogenesis

The molecular evidence of repetitive signalling throughout initiation and morphogenesis is reflected in the association of the anomalies of number, size and shape seen together clinically in the same dentition. This association has been validated by laboratory measurement of human dental study models.^49^

Hypodontia is associated with smaller tooth size that the average for the given population. The smaller teeth in the individual with hypodontia often also show morphological changes with reduced form seen, for example, as tapering of the crown of microdont lateral incisors and reduced cusp number and more rounded occlusal perimeter in molars (Fig. 1).

**Fig. 2:**
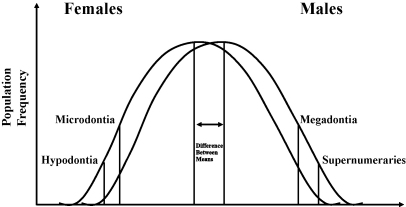
Underlying scale of continuous variation determining tooth size and number.

Hypodontia is a common condition in the population, present in some 25% of individuals. The aetiology within the population is multifactorial. Brook^48^ reviewed evidence of chromosomal, polygenic, single gene and environmental influences in this complex aetiology; different factors are a major influence in different individuals. Based on the gender differences in tooth size, frequency of anomalies of number and size and associations between them an aetiological model incorporating all the multifactorial influences was proposed^48^ (Fig. 2).

A number of recent studies have concentrated upon identifying mutations in some of the major single genes. Mutations in MSX1, PAX9, AXIN2 and EDA have been identified in families with non-syndromic hypodontia.^50–52^ Msx1 and Pax9 are co-expressed in dental mesenchyme at the bud and cap stages. Eighteen human mutations of PAX9 have been reported in hypodontia families, of which nine are missense. The DNA binding and transcriptional ability of Pax9 proteins are affected differently in each mutation.^53^ Missense mutations of Pax9 affecting the paired domain do not disrupt the physical interaction with Msx1 while in the G51S mutation synergistic co-operation with Msx1 is decreased or abolished in Bmp4/Luciferase assays. Functional regulation of Pax9 by homeobox proteins is necessary for early tooth development. The more severe clinical phenotypes correlate with mutations which result in the abrogation of DNA. In the G51S mutation the phenotype is moderate to severe hypodontia, yet there is intact DNA binding and increased transcriptional activation but lack of responsiveness to modulation by Msx1. In general the more severe phenotypes, with more congenitally absent teeth occur, relate to haploinsufficiency of Pax9 while those with mild or moderate hypodontia are associated with hypomorphic alleles.^53^ Differences in the patterns of hypodontia have been seen, with those having mutations of MSX1 showing particularly congenitally absent anterior teeth and those with PAX9 mutations hypodontia predominantly of posterior teeth. Mutations in MSX1 are also associated with oro-facial clefting, with and without hypodontia and with Witkop syndrome.^54–56^

The aetiological model reflects the well documented gender differences with females more often affected by hypodontia than males in a ratio of 1.5:1.^48^ Hypodontia, with small tooth size and reduced tooth form, usually occurs without systemic conditions. Occasionally it is seen as part of a syndrome such as ectodermal dysplasia.

The frequency of congenital absence of individual teeth also relates to their position in the different morphogenetic fields. In the permanent dentition the third molars, the second premolars, the upper lateral incisors and the lower central incisors are the most frequently absent teeth.^57^ These findings come from population and clinical multiple case studies. They probably reflect a general influence in hypodontia patients to reduced tooth tissue formation that results in smaller size and morphological changes in the earlier forming teeth in a field, i.e. affecting morphogenesis, while the later forming teeth at the end of the field have earlier effects on development, i.e. at initiation, and so fail to progress beyond the bud stage. While this is the general pattern in a population, in some individuals and families with hypodontia the pattern is different. In some the congenitally absent teeth may be concentrated in the anterior region while in others it is principally the molar region which is affected, reflecting mutations in such genes as PAX9 and MSX1.

The effect on tooth size in individuals having hypodontia is seen throughout the dentition. All teeth that develop in hypodontia subjects are smaller than those in control groups when measured by classical manual techniques^58^ and by image analysis.^58,89^ This is also in accord with the aetiological model proposed.^48,49^

An additional finding is that different dimensions of individual teeth, e.g. mesio-distal and bucco-lingual, are influenced to different degrees in hypodontia subjects compared to controls. Thus in a family with a mutation of PAX9 the mesio-distal dimensions were smaller to a different degree than the bucco-lingual dimensions of the formed teeth.^58^ A further finding in this PAX9 family is that different teeth were affected to different extents. The reduction in size was greatest in permanent canines in the hypodontia, PAX9 mutation, family members.^58^

Supporting evidence for the multifactorial nature of hypodontia comes from studies of families with severe hypodontia. In families with a member who had six or more missing permanent teeth, excluding the third molars, those family members with complete dentitions had teeth that were statistically significantly smaller than controls.^49,59^

Further evidence of regional influences within the dentition comes from a study of the distribution of congenitally absent teeth in 200 individuals with hypodontia. If one third molar is congenitally absent, the frequency of other third molars also being congenitally absent is much greater than expected by chance.^57^ Regional effects on tooth size were seen in four ethnic groups: while Chinese had the largest teeth overall, this effect was seen predominantly in the anterior regions and European and North American white Caucasians had larger molars than Chinese.^60^

In the contrasting anomaly of number to hypodontia, supernumerary teeth, contrasts in the patterns of the effects on the whole dentition occur. A series of population studies show males more frequently have supernumerary teeth than females as well as larger teeth than females.^48^ In patients with supernumerary teeth, the other teeth in the dentition are larger than those of controls.^61,62^ This difference is seen in the whole dentition, but there is a gradient effect on the degree of difference. Thus when the supernumerary tooth is in the upper central incisor region, the incisor teeth in the maxilla and mandible are the teeth that show the greatest difference. Image analysis measurement of the maxillary central incisors adjacent to the supernumerary show the effect on shape as well as size, having a more barrel shaped outline from the labial view than controls.

From studies of the mouse dentition it is suggested that mutations affecting Fgf, Eda, Bmp, Runx2, Apc, Shh and b-catenin are related to the occurrence of supernumerary teeth.^5,63–67^ Activation of b-catenin or ablation of Apc, an inhibitor of Wnt signalling, in embryonic mouse oral epithelium result in supernumerary teeth. The oral epithelium in adult mice remains responsive to b-catenin gain-of-function or APC loss-of-function and is still able to form new teeth.^53^ In the mouse supernumerary teeth may develop from vestigial tooth buds that are present in the incisor region and in diastema between the incisors and molars. These vestigial buds usually undergo apoptosis, but if degeneration by apoptosis does not occur a supernumerary tooth or element is formed.^68^ Supernumerary incisor and molar teeth as well as fused and large molar teeth develop in Lrp 4 and Wise deficient mice.^58^ Mice lacking the transcription factor Osr 2, odd-skipped related-2 develop supernumerary teeth lingual to their molar teeth of the normal series.^18^ Histological analysis traced initiation of these supernumerary teeth to aberrant thickening of the oral epithelium lingual to the first molar tooth buds.^18^ Expression of Pitx 2 and Shh was detected in the supernumerary dental placodes and expression of the dental mesenchyme markers Msx 1 and Lef 1 were upregulated and expanded lingually.^18^ Bmp 4 induces expression of ectodin whose inactivation was associated with fused first and second molars as well as supernumerary teeth in mice.^64,69^

Disruption of the antagonistic balance between Msx 1 and Osr 2 may underlie the hypodontia in Msx 1 null mice, the supernumerary teeth in Osr 2 deficient mice and the hypodontia in humans with Msx 1 mutations.^18^

Other important factors from mouse studies of the multilevel developmental process include the effects of upregulation and downregulation of specific genes and the results at a histological and clinical level on tooth number, size and shape. Changes in the regulation of Shh are related to holoprosencephaly, hypodontia and supernumerary teeth. Hypodontia and small size of teeth occur in mice that lack specific Eda-signalling pathway molecules.^68^

Under the control of Fgf/Bmp signalling, the homebox transcription factors Msx1 and Pax9 are expressed in the dental mesenchyme. In the absence of Pax9 the mesenchymal expression of Msx1, Lef1 and Bmp4 is downregulated and tooth development is arrested at the bud stage resulting in hypodontia.^10^ Loss of Eda leads to a smaller primary enamel knot and decreased expression of signalling molecules with the phenotypic outcome of smaller teeth with reduced cusp morphology and altered outline shape.^70^ Mouse studies also suggest Ectodin plays a major role in determining tooth size and shape. Ectodin, a Bmp and Wnt antagonist, integrates pro-apoptotic and pro-survival signals from the enamel knot and determines the area of the Bmp signal.^69^ Mice deficient in Ectodin show an increased area of Shh expression and large primary enamel knots, leading to large molars with a mesio-distal crest connecting cusps and supernumerary teeth.^64^ Supernumerary teeth and increased molar size and complexity are also seen with expression of an Edar receptor promoted by K14.^71^

The relationship between development and anomalies of number, size and shape of teeth in modern human studies reflects the reiterative patterns seen in mouse models of signalling of the ectodermal–mesenchymal interactions during tooth germ initiation and morphogenesis. Epigenetic events relating to the spatial arrangement of cells and the timing of the interactive signalling may explain differences in tooth number, size and shape and dental asymmetry in monozygotic twins.^72^

Similar findings to those for modern human populations for tooth number, size and shape were gained in studies of Romano-Britons. Females had smaller teeth than males and had a higher frequency of hypodontia and microdontia: males had a higher frequency of supernumerary teeth and megadontia,^73,74^ The teeth of the Romano–Britons were smaller than modern Britons, possibly reflecting major environmental effects.^73^ These findings are compatible with the multifactorial model and provide an example of the interaction of genetic and environmental factors.

### 3.3 Anomalies of tooth structure occurring during differentiation and biomineralisation

As the developmental process continues a marked change occurs from the determining of the tooth germ as one of a series of structures, its position in the jaws in relation to other tooth germs and its size and shape, to its definition as a unit consisting of different hard and soft tissues each contributing to its function as an organ.

#### 3.3.1 Developmental defects of dentine

In the multilayered process the first hard tissue to differentiate and commence mineralisaiton is dentine. Clinically, inherited anomalies in man have been classified as three types of dentinogenesis imperfecta and two types of dentine dysplasia. The inheritance patterns of these families are autosomal dominant. The clinical and radiographic appearance varies not only between the different anomalies of dentine, but also between affected family members with the same mutation, between the primary and permanent dentitions in an affected individual and even between teeth in the same dentition.

At a tissue level, as differentiation commences the odontoblasts secrete the dentine matrix which, while mainly collagen, also includes proteins from the Dspp gene. At the molecular level dentinogenesis imperfecta and dentine dysplasia have been related to non-functional variants of Dspp in a mouse model.^75^ Transgenic mice overexpressing TgfB1 under the upstream regulatory sequence of Dspp have teeth with decreased mineralisation and abnormal formation of dentine, resembling those of patients with dentinogenesis imperfecta and dentine dysplasia.^32^ Pleiotrophin has a critical role in dentinogenesis: Ptn knock out mice have a dentine phenotype with enlargement of pulp chambers and decreased mineralisation of dentine.^31^ Anomalous collagen in Ehlers–Danlos syndrome has been related to the dentine abnormalities found in these patients.

#### 3.3.2 Developmental defects of enamel

This group of dental anomalies is common in the population. The large majority of enamel defects are isolated to the dentition but they are also described in patients with syndromes. The aetiology of developmental defects of enamel is complex and in the population is multifactorial. The major single gene defects are uncommon while a number of important environmental factors have been substantiated.

##### 3.3.2.1 Prevalence

Using the F.D.I. D.D.E Index (1982) studies of White Caucasians in U.K., Ireland and New Zealand found that in each population 60–65% of individuals had at least one enamel defect.^76^ Approximately 10% of a U.K. sample had 10 or more teeth affected.^77^ In molar incisor hypomineralisation a series of studies have shown frequencies of 3–19% in different populations.^78,79^ Hereditary enamel defects have reported frequencies from 1:700 to 1:14,000.^80,81^ Some of this variation may well reflect the inclusion, or not, of affected large families in the sample.

A new index for the accurate clinical scoring of enamel defects, the E.D.I. index, has been developed.^82^ This has a simple backbone and digital scoring leading to a high degree of reproducibility. The index has been further developed for more detailed scoring of subtypes of defects when this is indicated.^83,84^

##### 3.3.2.2 Amelogenesis imperfecta

Hereditary enamel defects, as hereditary dentine defects, occur as anomalies of the dentition alone and as part of syndromes. The term, amelogenesis imperfecta, often has been used for the former, but is sometimes applied to both.

Clinically, amelogenesis imperfecta presents in hypoplastic, hypocalcified and hypomature forms. The hypoplastic types vary from the absence of much of the enamel to areas of pits and grooves distributed in patterns over the tooth surface. The hypoplastic enamel is hard when probed. The hypocalcified variety presents as a full thickness of enamel with opacities, sometimes with varying appearance over different areas of the surface. Post-eruptive breakdown of the soft enamel readily occurs.^85^ The hypomature types also have a full thickness of enamel and present as opacities, varying in colour between the different types. In the X-linked varieties clinical appearance differs between the genders. In males the hypoplasia affects the whole surface while in females these are alternating vertical bands of normal and hypoplastic enamel. Similar effects are seen in X-linked hypomature defects.

Findings concerning the clinical and histological appearance raise interesting questions about the expression of the single gene mutations affecting the enamel. First, antimere teeth are not always mirror images. For example, in the females with X-linked amelogenesis imperfecta the pattern of vertical banding may vary between the corresponding teeth on left and right sides of the arch. Secondly, the anomalous enamel in some individuals is not found throughout the whole thickness of the enamel. For example, in some exfoliated primary teeth from children with amelogenesis imperfecta no surface lesion could be detected visually or by image analysis, but marked sub-surface lesions in the enamel were demonstrated histologically.^84^ Thirdly, different areas of the tooth surface, e.g. incisal and occlusal portions compared to cervical areas, may show different appearances of enamel even in varieties determined by autosomal dominant inheritance.^85^ As noted in inherited dentine defects, the appearance varies not only between the different anomalies but also between family members with the same mutation, between the primary and permanent dentition in an affected individual and even between teeth in the same dentition.^86^

The hypoplastic, hypocalcified and hypomaturation types arise at different stages of amelogenesis emphasising again the importance of the fourth dimension of time in the developmental process. Another marked clinical finding is that in many patients a mixture of different types of defect occurs and therefore the current classifications of amelogenesis imperfecta types are based on the predominant appearance in that individual and family. The hypoplastic types relate to secretory defects, the hypocalcified to mineralisation defects and the hypomaturation to late protein processing and crystallite maturation defects.^87–89^

To date specific types of amelogenesis imperfecta have been described having X-linked, automsomal dominant and autosomal recessive modes of inheritance.^90^ X-linked amelogenesis imperfecta has been linked to mutations in the amelogenin gene (AMELX) located at Xp22.3–p22.1. Amelogenin is also encoded on chromosome Yp11^91^ and mutations at different sites have been described.^92^ Different mutations in AMELX have allowed initial correlations between genotype and phenotype.^93^ Signal peptide mutations causing a total loss of amelogenin can be associated with hypoplastic enamel defects. Missense mutations have been associated with hypomineralisation and C terminus mutations with hypoplastic defects.^93^ Wright et al.^94^ identified three different AMELX mutations. One showed marked hypoplasia with hypomineralisation, the enamel lacking prismatic architecture and females having areas of more normal enamel adjacent to regions of more affected enamel. The other two mutations were associated with enamel of near normal thickness with a prismatic structure and decreased mineral content apparently due to maturational defects.

Some types of autosomal dominant hypoplastic amelogenesis imperfecta has been associated with mutations in the enamelin gene (ENAM); eight mutations have been identified.^95^ Individuals homozygous for the g13185_13186insAG ENAM mutation had hypoplastic enamel pitting with an anterior open bite, while those who were heterozygous for this mutation presented with only enamel pitting.^96^ Enamel phenotypes of ENAM mutations may be dose dependent with generalised hypoplastic amelogenesis imperfecta segregating as a recessive trait and localised pitting as a dominant trait.^86^

Two ENAM mutations^94^ were associated with generalised thin enamel that had a rough surface and lacked prismatic structure, having a laminated type of pattern. The developmental mechanism in humans with ENAM mutations associated with localised pitted hypoplastic defects probably differs from that causing generalised hypoplasia in the Enam null mice.^86,94,96,97^ Indeed in Enam null ^−^/^−^ mice it was reported that there was no true enamel, ectopic amelogenin secretion and ectopic mineralisation.^42^ Humans with ENAM mutations that cause haploinsufficiency present with localised hypoplasia, while generalised hypoplasia is probably related to a dominant negative effect.

Ameloblastin deficiency results in severe enamel hypoplasia with lots of prism structure in mouse models, although no human mutations have yet been described in amelogenesis imperfecta patients.^94^ In the absence of ameloblastin, Msx2, p27 and p75 are deregulated leading to ameloblasts reverting to an undifferentiated state.^98^ Ameloblastin overexpression in a transgenic mouse results in a phenotype resembling amelogenesis imperfecta.^99^

For some other genes encoding for enamel matrix proteins, e.g. ameloblastin (AMBN 4q 11), tuftelin (TUFT1 1q 21) and amelotin (4q 13.3) mutations have not yet been identified in patients with amelogenesis imperfecta.

Human mutations in genes encoding for enamel proteinases, MMP20 and KLK4, are associated with apparently normal enamel thickness but have varying degrees of hypomineralisation.^100–102^ Mice lacking expression of Mmp20 have hypoplasia coupled with hypomineralisation. In human autosomal recessive pigmented hypomaturational amelogenesis imperfecta, although MMP20 is expressed throughout the secretory stage and into early maturation while KLK4 expression only starts in transition/early maturation and continues to tooth eruption,^45^ the MMP20 and KLK4 mutations reported to date have similar clinical phenotypes with normal thickness enamel which is pigmented and soft.^92^ In a Klk4 null mouse the retention of enamel proteins within the enamel at the maturation stage prevented full maturation of the enamel crystallites, reducing their growth.^103^

Using the main candidate genes in families with amelogenesis imperfecta less than one quarter were found to have mutations in these genes.^94,104^ Some genes which do not encode for enamel proteins have now been identified as having mutations in certain amelogenesis imperfecta patients. Families with autosomal dominant hypocalcified amelogenesis imperfecta (ADHCAI) have been identified with mutations in the FAM83H gene (8q24.3). The enamel is of normal thickness but has a marked decrease in mineral content.^94^ The mutations segregated with the clinical phenotype, demonstrating that FAM83H is required for enamel calcification.^105^ Four nonsense and two 2 bp deletion FAM83H mutations have been identified.^106^ The presence of skeletal class III malocclusion and craniofacial form deviation from normal was much more prevalent in the affected individuals compared with unaffected family members. Those with generalised ADHCAI had normal prismatic architecture with decreased mineral content and increased protein content. A localised phenotype where the cervical enamel was predominantly involved has also been described.^94^ The genotype–phenotype relationships reported were that mutations producing proteins of 677 amino acids or less presented with a classic ADHCAI phenotype while those producing proteins of 694 or more amino acids has the localised, predominantly cervical abnormal enamel.^94^ In a family with amelogenesis imperfecta hypoplastic-hypomaturation with taurodontism (AIHHT) which was autosomally dominantly inherited, a mutation is reported in the homeodomain of DLX3 (17q21).^107^

Comparison of human and mouse genotype–phenotype relationship has shown much similarity but some differences. While many human mutations alter function of the normal protein product, most mouse models for amelogenesis imperfecta candidate genes are designed to knock out the gene.^94^ These null models do not reflect fully the human gene mutations and the more diverse human genetic background, including possible epigenetic interactions.

##### 3.3.2.3 Syndromes

The presence of enamel defects in patients with syndromes may provide further candidate genes as suggested by the mutations in Dlx3 that cause tricho-dento-osseous syndrome. In TDO the dental defects are enamel hypoplasia and taurodontism. The severity of the enamel defects varies from very thin enamel to slightly decreased enamel thickness.^108^ While one study suggested that TDO and amelogenesis imperfecta with taurodontism (AIT) are genetically distinct^108^ a later study suggested that TDO and AIT are allelic for Dlx3.^107^

Generalised developmental defects of enamel have been described in two first cousins who also had hypohidrosis but no other observed ectodermal defects.^109^ In Jalili syndrome, consisting of autosomal recessive cone-rod dystrophy and amelogenesis imperfecta, mutations in the CNNM4 gene (2q11) have been identified.^110^ Nine mutations are described in all, three missence, three terminations, two large deletions and a single base insertion. In addition many other syndromes present with defects of dentine and enamel structure, including tuberous sclerosis, epidermolysis bullosa, the lipidoses and mucopolysaccharidoses.^25,111^

In some syndromes the enamel defects frequently present appear to be a secondary cause of the primary disease. In autoimmune polyendocrinopathy–candidiasis–ectodermal dystrophy (APECED), enamel hypoplasia is seen as horizontal bands or generalised pitting in 80% of the patients.^112^ Patients with vitamin D-resistant rickets (VDRR) may show enamel defects with a chronological distribution; these defects relate to periods before the condition has been diagnosed and adequately treated.^111^ In Coeliac disease enamel defects with a distribution pattern corresponding to the sequence of tooth development may be seen in primary and permanent teeth, again depending on the time of diagnosis and successful treatment, including compliance, of the disease.^113^

##### 3.3.2.4 Environmental factors

Enamel defects are recorded as arising from localised or generalised insults to the developing dentition.

Local enamel opacities or hypoplasia may arise from trauma to the permanent tooth such as opacities or germ. This usually occurs by intrusive luxation or avulsion of the primary tooth associated with a fall in a young child, but may result from ritual dental mutilation. Similarly a long lasting periradicular infection of a primary tooth may result in a range of developmental disturbances of the permanent successor, varying from enamel opacities to enamel hypoplasia and to arrest of development of the permanent tooth germ.^114^ Which of these various anomalies occur is affected by the interaction of such factors as the timing of development, the severity and duration of the insult and the host's susceptibility and response.^114^ Therapeutic irradiation can severely disturb the development of teeth lying in the path of the radiation beam, resulting in enamel defects, microdontia, arrested root development and absence of some teeth.

Generalised defects of the dental hard tissues of systemic origin can occur prenatally, perinatally, postnatally, during infancy, or during early childhood. At birth, even the normal change from intrauterine to extrauterine life may have an adverse effect on amelogenesis and dentinogenesis as evidenced by the so-called neonatal line. Any stressful event during birth is likely to accentuate this line, resulting in clinically evident enamel defects.^115^

Other factors associated with developmental defects include: ingestion of chemicals (fluorides, tetracyclines, dioxins, thalidomide); prematurity/low birth weight; severe malnutrition, neonatal hypocalcemia, vitamin D deficiency; bilirubinemia, thyroid and parathyroid disturbances; maternal diabetes; neonatal asphyxia; severe infections; and metabolic disorders.^76,116^

The resultant defect is not specific to the generalised environmental insult but depends, as with localised defects, on the timing, severity and duration of the insult, the stage of development of the dentition and the host's susceptibility; the same insult results in different responses in different individuals. There are two important clinical consequences of this: only rarely can an aetiological diagnosis be made from the clinical appearance and the defects have a distribution reflecting the stage of dental development (the chronology) at which they occurred.

#### 3.3.3 Defects of uncertain aetiology

Some developmental defects do not present with patterns that can be related to those patterns seen in conditions of known aetiology. Regional odontodysplasia is a rare developmental anomaly affecting all the dental hard tissues in a segment of the dentition.^117^ There is some evidence to suggest that the pathogenesis involves vascular deficiencies during development, while another possibility is a somatic mutation.^118^

Molar–incisor hypomineralisation is the term given to enamel opacities of presumed systemic origin affecting one or more first permanent molars and some or all permanent incisors. It has a high frequency, affecting between 4% and 17% in different studies in a range of populations. The lesions vary in extent from small demarcated white, yellow or brown opacities to those covering much or the entire crown. The aetiology remains unclear.^119^ The defects appear to be related to altered ameloblast function during the transitional and maturation phases. A family history of enamel defects is more frequently reported in children with MIH than controls, but this association has not been found to be statistically significant.^119,120^ Systemic environmental insults have been explored in a number of studies. Among possible toxins various polyhalogenated aromatic hydrocarbons, dioxins and dibenzofurans may disturb tooth development.^121^ MIH was significantly more common among children of mothers who had health problems during pregnancy.^119^ A medical problem related to birth, e.g. premature birth, prolonged delivery, cyanosis, was found frequently in one group of affected children.^122^ The developmental stage of the ameloblasts at the time of the insult may be important, with the transitional stages of enamel maturation being particularly susceptible to such insults as infection with varicella zoster virus.^119,123^

MIH often occurs asymmetrically and so differs from ‘chronological hypoplasia’, which usually has symmetrical defects and an identifiable systemic environmental insult. MIH may have a multifactorial aetiology with a genetic predisposition associated with one or more of a range of systemic insults occurring at a susceptible stage in the development of specific teeth. Problems remain when in a seeming random manner several teeth are severely affected while their antimeres are unaffected. Interestingly there is also an irregular pattern of enamel defects sometimes in patients with epidermolysis bullosa.^111^

#### 3.3.4 A unifying model for the aetiology of enamel defects

As reviewed above many specific causes of enamel defects, genetic and environmental, have been identified. Genetically these include mutations and deletions of single genes, both those affecting the oral tissues alone and those related to more widespread syndromes. Some enamel defects may also be due to multifactorial inheritance.^77^ Environmental factors may be local or systemic and their effect depends on the stage of development of each tooth, the severity and duration of the insult and the host's susceptibility.^114^

The complexity of the aetiology is emphasised by the gene–gene and gene–environment interactions already considered. In both human and mouse studies, for example, there is considerable individual and strain variation in enamel defects in response to a standardised fluoride intake. The complexity of the clinical situation extends to considering why, for example, some permanent incisors, presumably developing simultaneously, have enamel defects and others do not in such conditions as molar–incisor hypomineralisation and epidermolysis bullosa.

**Fig. 3:**
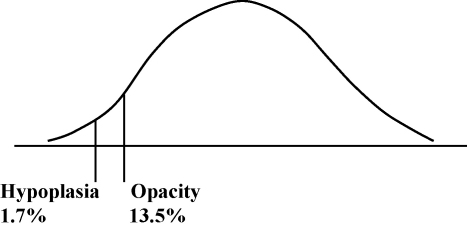
Tooth prevalence of enamel defects – curve represents outcome of multifactorial aetiology.

To interpret these findings a model is proposed based on a multifactorially determined continuous distribution with thresholds delineating those affected^124^ (Fig. 3). The location of an individual in this distribution is determined by the interaction of the factors outlined above.

## 4 Conclusions and forward

The findings reviewed above illustrate that the aetiology of dental defects is multifactorial and requires the dental developmental process to be considered on multiple levels, in multiple dimensions and as a progression over time.

### 4.1 Multifactorial

It is multifactorial with various genetic, epigenetic and environmental factors. The interactions and outcomes of these factors during development are complex. Even when a specific mutation of a single gene or one major environmental insult has been identified, detailed investigation of the phenotype often reveals variation between affected individuals in the same family, between dentitions in the same individual and even between different teeth in the same dentition.

Mutations and deletions at different points in the same gene result in different phenotypes, as seen with AMELX and DSPP genes. The phenotype seems to depend not only on the severity of the genetic defect but more on the site of the change and its effect on the protein product. The same, or closely similar phenotypes, whether anomalies of tooth number or structure, may arise from different aetiologies: not only mutations in different genes but also environmental factors may result in similar phenotypes.^116^ Related to the action of a number of the developmental regulatory genes active in odontogenesis, in different tissues, mutations can result in syndromes of which dental anomalies are part. A different causation is at work when the developmental dental defect is secondary to the systemic effects of the syndrome as seen in the enamel defects in patients with Coeliac disease or vitamin D-resistant rickets.

Further factors to be considered in this multifactorial aetiology are chromosomal anomalies; epigenetic effects, both ‘narrow’ effects on DNA and broader alterations in gene expression; and environmental effects, both general, long term (as nutritional deficiencies) as major, short term insults.

In view of this multifactorial nature of dental anomalies, in future, in addition to seeking specific mutations of single genes, the complex background within which they are acting should be explored. The antagonistic balance between developmental regulatory genes, acting as activators or inhibitors of odontogenic potential, results in the normal phenotype of tooth number, size and form. If the positive feedback loop between genes in the dental mesenchyme is disrupted hypodontia or supernumeries can result. If the normal balanced control of Shh activity is upregulated or down regulated, dental anomalies occur. The signalling pathways of which the single gene is part are important as well as their interactions and the possibility of compensation by substitutions or recovery. Further investigations are needed to identify new genetic loci associated with specific anomalies, e.g. amelogenesis imperfecta. In addition further genotype–phenotype investigations are required in which the phenotype is accurately recorded and measured exploiting the new 2D and 3S techniques available.^125^

### 4.2 Multilevel

The events and outcomes of dental development occur and present at three different levels, which need to be collated to increase understanding.^126^ Molecular and cellular interactions, both intracellular and extracellular determine the macroscopic phenotype. Future studies need to collate findings at each of these levels, fully defining the phenotype, both human and murine.

### 4.3 Multidimensional

The molecular and cellular interactions and their outcomes are multidimensional. The *x*, *y* and *z* axes are affected to different extents by specific genes, resulting in the morphology of the individual tooth. In addition there is a precise spatio-temporal pattern in the sequential initiation, morphogenesis and differentiation of tooth germs. Antagonistic signalling controls expression and influences the spacial distribution of odontogenic signals in morphogenetic fields. To form the dentition, apoptosis within enamel knots plays an important role in regulating tooth size and shape. Different tooth germs are at different stages of development at any one time point and therefore a substantial disruption, e.g. a major environmental insult, over a defined chronological period may cause different defects in different teeth depending on the developmental stage of the individual tooth germ.

The clinical phenotype reflects these spatio-temporal effects. The tooth size of the whole dentition is influenced when variations in tooth number occur, but within the dentition there is a gradient with the greatest effect on size being close to the site of the anomaly. The variations of number, size and shape most frequently occur at the boundaries of morphogenetic fields. Considering particular tooth types the congenital absence of one third molar is associated with an increased risk of absence of its antimere as well as the third molars in the opposing arch. Related to these findings is the work in animal models on the spatial distribution of specific genes around the arch.

### 4.4 Progression over time

Time is another important dimension in the normal and abnormal development of the dentition. Different genes control different stages of the developmental process. The activity of genes is switched on and switched off. Transient signalling centres in the enamel knots have been identified, with apoptosis following cessation of signalling. There are critical stages in the development of individual tooth germs and, if progression fails, the germ will not develop further and may undergo apoptosis. Some of the transcription factors necessary for progression have been identified and different members of the same family of factors may compensate when one is inactivated.

The reiterative signalling patterns over time during the sequential process of initiation and morphogenesis^127^ are reflected not only in the variation of molar cusps but also in the clinical presentation of the association of anomalies of number, size and form.^48^ This association, proposed from a series of studies has been modelled (Fig. 2) and further tested, confirming its validity.^48,58,59,61,62^ In the future human and murine studies of anomalies of tooth number, full clinical phenotyping including the accurate determination of tooth size and shape is needed to relate the phenotype outcome to the multifactorial developmental process and allow further modelling. To link phenotype to the genotype in mammalian teeth a mathematical model has been developed integrating experimental data on gene interactions and growth.^128^

**Fig. 4:**
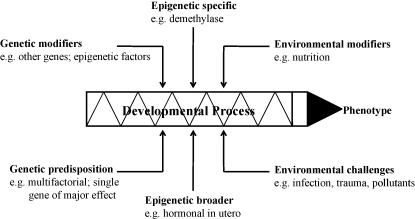
Summary diagram for aetiology of dental anomalies.

Time also is a factor within each morphogenetic field with the later developing tooth being smaller, less complex in shape and showing greater variability. Moreover in patients with hypodontia, not only is there reduced tooth size and form compared to controls, but the teeth erupt later^48^, showing that the whole developmental process is affected. A diagram to summarise visually the various inputs to the aetiology of dental anomalies is given in Fig. 4.

## Disclosures

*Competing interests:* None declared.

*Funding:* This research was supported by Wellcome Trust.

*Ethical approval:* Not required.

## Figures and Tables

Upper part of figure reproduced from http://bite-it.helsinki.fi/.
